# Evaluating the impact of disability support services on healthcare utilization in individuals with disabilities and hypertension in Korea

**DOI:** 10.1038/s41598-025-86915-x

**Published:** 2025-04-24

**Authors:** Mingee Choi, Woorim Kim, SungKyung Park, Junbok Lee, Jaeyong Shin

**Affiliations:** 1 Department of Preventive Medicine, Yonsei University College of Medicine, 50 Yonsei-ro, Seodaemun-gu, Seoul, 03722 Republic of Korea; 2 National Hospice Center, National Cancer Control Institute, National Cancer Center, Goyang-si, Gyeonggi-do Republic of Korea; 3 Division of Cancer Control & Policy, National Cancer Control Institute, National Cancer Center, Goyang-si, Gyeonggi-do Republic of Korea; 4 Department of Bigdata AI Management Information, Seoul National University of Science and Technology, Seoul, South Korea; 5 Institute for Innovation in Digital Healthcare, Yonsei University, Seoul, Republic of Korea

**Keywords:** Disability, Hypertension, Health policy, Hospital utilization, Chronic disease management, Health care, Medical research

## Abstract

People with disabilities often face heightened barriers to accessing healthcare services, resulting in unmet medical needs and increased prevalence of chronic conditions such as hypertension. In response, the Korean government expanded the Personal Assistance Service (PAS) program to enhance healthcare accessibility and support daily living needs for individuals with disabilities. This study aims to evaluate the impact of the PAS program expansion on healthcare utilization, specifically focusing on hospital visits for hypertension management among people with severe disabilities. We used sample cohort data from the National Health Insurance System. The difference-in-differences approach was used to estimate differences in hospital utilization between the treatment and control groups before and after the intervention period. The expansion of PAS program was associated with an increase in hypertension total visits increase of 0.128. Specifically, the number of outpatient visits increased by 0.132. The increase in hospital visits was more pronounced in unclassified disability types. Seasonal variations and higher Charlson Comorbidity Index (CCI) scores were positively associated with increased hospital visits. The results reveal that expanding enrollment in programs for people with disabilities was linked to an increase in outpatient visits among people with severe disabilities. This study is noteworthy because the expansion of services could potentially help bridge health disparities.

**Supplementary Information:**

The online version contains supplementary material available at 10.1038/s41598-025-86915-x.

## Introduction

Vulnerable health conditions in people with disabilities can cause chronic diseases earlier than in non-people with disabilities, leading to secondary dysfunction^1,2^. Previous studies have indicated that people with disabilities are more likely to have chronic diseases than non-people with disabilities, and this is also the case in South Korea (hypertension, 62.8% versus 56.2%; diabetes, 25.7% versus 18.8%)^3–7^. People with disabilities have greater barriers to using medical services for physical, social, and economic reasons^8,9^. In a national survey on the status of people with disabilities, the rate of “unmet medical care” decreased to 22.4% and 15.4% in 2009 and 2017, respectively^10,11^.

The Korean government has provided the Personal Assistance Service (PAS) program for people with disabilities to improve medical accessibility^12^. The program provides financial support for activity assistance, including mobility assistance (hospital visits, social activities), physical support (personal hygiene, meal preparation), and domestic support (cleaning, cooking). It also includes home-visit bathing and home-visit nursing services, which offer specialized nursing care, health consultations, and oral hygiene services. Eligible beneficiaries include all registered individuals with disabilities aged between 6 and 65 years. This subsidy can increase their hospital visits by covering long-distance travel and providing assistance from social workers or nurses. In addition, home-visit nursing can indirectly help enhance medical accessibility for people with disabilities by encouraging hospital visits when issues arise.

Initially implemented in 2010, the program targeted only the most severely disabled individuals (Grade 1). By January 2013, the eligibility was expanded to include both Grade 1 and Grade 2 (severe disabilities), and by June 2015, some Grade 3 (mild disabilities) were also included. Following the abolishment of the disability grading system in July 2019, the program further expanded to cover all registered individuals with disabilities, regardless of their previous grade.

The evolution of the PAS Program has focused on improving healthcare access and supporting the daily living needs of people with disabilities, ultimately aiming to enhance their quality of life. Prior studies provide indirect evidence that financial and social support interventions can lead to increased healthcare utilization, particularly among individuals with chronic conditions such as hypertension^13,14^. However, these studies have confirmed that support for vulnerable populations influences healthcare utilization but have not directly verified whether financial interventions for individuals with disabilities improve healthcare accessibility. Given that hypertension is a leading risk factor for cardiovascular diseases and that its prevalence is higher among people with disabilities, the provision of economic and social support through this program may have contributed to increased hospital visits for hypertension management. In the case of hypertension, which has been reported as the most common risk factor for cardiovascular disease worldwide, the prevalence of people with disabilities is higher than that of the general population^15^.

Therefore, the purpose of this study is to assess the impact of the expansion of the PAS program on hospital visits among people with disabilities who have hypertension. This study aims to evaluate how the program’s expansion has contributed to improved chronic disease management and enhanced access to healthcare services for people with disabilities.

## Methods

### Study design

**Fig. 1:**
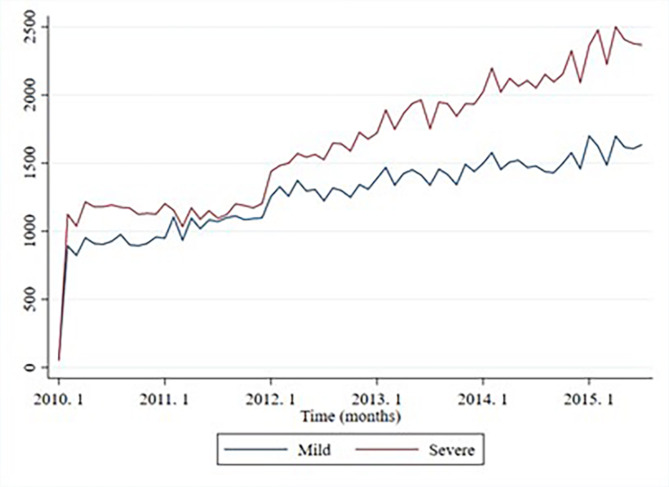
Unadjusted trend in hospital visits due to hypertension.

This study aimed to examine the changes in medical utilization after the expansion of the national disabled support service in Korea using a difference-in-difference (DID) approach. As the national disabled support service expanded the implementation of services for people with severe disabilities, starting on January 1, 2013, the pre-and post-intervention periods were from January 1, 2010, to December 31, 2012, and January 1, 2013, to May 31, 2015, respectively. A prerequisite for DID estimation is that the trends in the test and control groups should be parallel, which can be partially verified using graphical evidence^16^. Figure 1 and Supplementary Table 1 shows that the treatment (severely disabled) and control (mildly disabled) groups satisfied the basis for a common trend.

### Data

This study used sample cohort data from the National Health Insurance Service (NHIS). The data contained socioeconomic qualification variables (including death and disability), the status of medical resource utilization, and the clinical status of approximately one million people. The data consists of a stratified sample representing 2% of the entire national population, stratified by gender, age, and region, and is derived from health insurance claims data. The classification of severe and mild disabilities was available as a sociodemographic variable in the dataset. The study population comprised individuals with disabilities and hypertension (ICD-code: I10–I15) between 20 and 64 years old (PAS program service target). The total number of observations were 17,126, recorded from January 1, 2010, to May 31, 2015. The final study sample consisted of 38,499 individuals, including 15,103 in the treatment group and 23,396 in the control group.

### Variables

In this study, the main outcome variable was defined as the number of hospital visits due to hypertension during the entire observation period, including both pre- and post-intervention. This outcome was selected to assess changes in hospital utilization associated with hypertension before and after the intervention. The independent variables included treatment status, gender, age, health insurance type, disability type, the Charlson Comorbidity Index (CCI), and a time variable.

The treatment variable distinguishes between the treatment (severely disabled) and control (mildly disabled) groups allowing for the evaluation of intervention effects. Gender and age were included to capture demographic characteristics, with age treated as a continuous variable. Health insurance type was categorized into employer-sponsored, local subscribers, and Medical Aid recipients. Disability type was classified into physical disabilities, brain lesions, visual impairment, hearing impairment, and other disabilities. The Charlson Comorbidity Index (CCI) was incorporated as a measure of comorbidity to adjust for the severity of concurrent health conditions. Lastly, a time variable was included to distinguish the periods before and after the intervention, which is essential for the Difference-in-Differences (DID) analysis used to estimate the causal effect of the intervention.

### Statistical analysis

In this research, the DID methodology is employed to estimate the causal effect of policy interventions by analyzing changes in outcomes across different groups. The DID approach controls for unobserved time-invariant confounders by comparing the difference in outcomes before and after the intervention for the two groups. By modeling the pre-intervention outcome, an appropriate counterfactual is established, representing what would have occurred in the absence of the policy intervention. To estimate the average treatment effect, given the outcome variable is count-based, a negative binomial distribution model is utilized. The negative binomial model includes an additional parameter to handle this overdispersion, making it more suitable for modeling count data compared to a Poisson regression. This ensures a more accurate estimation of the effect size while accounting for the inherent variability in the data. The model incorporates key covariates such as age, gender, severity of disability, and comorbidities to control for potential confounding factors. Interaction terms between the treatment group and the post-intervention period are included to estimate the DID effect, representing the average treatment effect. All analyses were conducted using the SAS software, version 9.4 (Cary, NC, USA).

## Results

### Characteristics of study participants

Table 1 provides a detailed comparison of the general characteristics of study participants, including 38,499 individuals (15,103 in the treatment group and 23,396 in the control group). For hypertension patients, the average number of inpatient visits showed a slight increase in both groups (from 1.5 to 1.7 in the treatment group and from 0.5 to 0.7 in the control group).

**Table 1:** Characteristics of study participants.

*N* = 38,499				
	Treatment Group (severe) *N* = 15,103		Control Group (mild) *N* = 23,396	
	Pre (2010–2012)	Post (2013–2015.5)	Pre (2010–2012)	Post (2013–2015.5)
Number of hypertension visits				
Inpatient, mean (SD)	1.5 (5.1)	1.7 (7.7)	0.5 (2.3)	0.7 (3.1)
Outpatient, mean (SD)	27.3 (58.9)	30.6 (64.9)	18.5 (25.7)	17.0 (20.6)
Social demographic				
Women, %	28.2	33.9	25.9	28.5
Type of insurance, %				
Employer-sponsored	30.8	34.9	50.9	55.9
Local-subscriber	29.7	26.6	36.5	32.7
Medical care	39.5	38.5	12.6	11.3
Type of disabilities, %				
Physical	30.8	31.1	70.4	66.8
Acquired brain injury	15.1	13.7	5.5	7.4
Vision impairment	3.9	3.4	11.4	11.9
Deaf or hard of hearing	5.1	5	3.9	3.5
Etc.*	45.1	46.8	8.8	10.4
Age, %				
20s	0.8	1.1	0	0.1
30s	2.3	3.6	0.8	0.8
40s	9.8	9.9	5.5	7.0
50s	36.7	37.2	32.8	33.9
60–64	50.0	48.1	60.9	58.2
Age, mean (SD)	59.4 (5.5)	56.8 (7.9)	59.4 (5.5)	59.0 (5.8)
CCI, mean (SD)	1.4 (0.7)	1.6(0.88)	1.4 (0.7)	1.6(0.85)

*Unclassified disabilities included intellectual disabilities, kidney disorders, mental disorders, kidney disorders, speech disorders, and autism.

However, outpatient visits increased significantly in the treatment group (from 27.3 to 30.6) and slightly decreased in the control group (from 18.5 to 17.0) after the expansion of the PAS program. The demographic characteristics, such as sex and age, remained similar between the treatment and control groups before and after the intervention. Differences were observed in health insurance type and disability type. The proportion of physical disabilities was highest in the control group, while unclassified disabilities were most common in the treatment group. The employment-sponsored insurance rate was lower in the treatment group, while the local-subscriber rate was also lower compared to the control group.

## Trends in the prevalence of disabled-hypertension

Figure 1 shows the trends in the total number of hospital visits due to hypertension for both the treatment (severe disability) and control (mild disability) groups from 2010 to 2019. Overall, there was a steady increase in hospital visits over time in both groups. Notably, the increase in visits among individuals with severe disabilities accelerated after 2013, coinciding with the expansion of the support program for people with disabilities.

## Effect of national policy on behavior of people with disability and hypertension

Table 2 shows the effect of national support services for people with disabilities using a DID estimate with adjustment for all covariates. The expansion of PAS program was associated with a significant increase in the total number of visits (0.128 per month, *p* < 0.05). Specifically, there was an increase in monthly outpatient visits by 0.132 (*p* < 0.05). Seasonal effects were also observed; outpatient visits were significantly higher in all seasons compared to winter. Regarding disability types, unclassified disabilities had significantly more hospital visits compared to other types. The Charlson Comorbidity Index (CCI) showed a positive association with the number of visits, indicating that patients with higher comorbidity had more frequent hospital visits.

**Table 2:** Effect of national policy on behavior of people with disabilities and hypertension.

	Average change of total visits		Average change of outpatient visits		Average change of inpatient visits
Intercept	1.865*** (0.104)		1.748*** (0.133)		− 0.166 (0.368)
Case (REF: Mild)	0.151*** (0.035)		0.138** (0.038)		0.335** (0.106)
Policy (REF: Control)	− 0.09** (0.029)		− 0.103** (0.031)		0.009 (0.090)
Policy*Case (REF: Control, Mild)	0.114** (0.043)		0.111* (0.047)		0.149 (0.131)
Type of insurance (REF: Medical Care)					
Local-subscriber	− 0.048 (0.03)		− 0.001 (0.032)		− 0.641*** (0.087)
Employer-sponsored	− 0.026 (0.028)		0.003 (0.030)		− 0.991*** (0.081)
Sex (REF: Male)	0.010 (0.024)		0.020 (0.026)		− 0.127 (0.073)
Age	0.007** (0.002)		0.007** (0.002)		0.012 (0.005)
Season (REF: Winter)					
Spring	1.167*** (0.041)		1.191*** (0.044)		0.546*** (0.125)
Summer	0.304*** (0.047)		0.305*** (0.050)		0.256 (0.143)
Autumn	0.195** (0.053)		0.165** (0.057)		0.505** (0.159)
Type of disability (Ref: etc.)					
Physical	− 0.484*** (0.031)		− 0.479*** (0.033)		− 0.804*** (0.091)
Acquired brain injury	− 0.506*** (0.042)		− 0.674*** (0.045)		0.984*** (0.115)
Vision impairment	− 0.443*** (0.046)		− 0.434*** (0.049)		− 0.798*** (0.143)
Deaf or hard of hearing	− 0.504*** (0.057)		− 0.496*** (0.062)		− 0.804*** (0.182)
CCI	0.167*** (0.013)		0.169*** (0.014)		0.126** (0.041)

The robust standard error from clustering to the district level is shown in parentheses. *, **, and *** denote significance at the 10%, 5%, and 1% levels, respectively.

## Discussion

This study aimed to confirm whether the number of outpatient days for people with severe disabilities and hypertension increases through the Activity Support Program. The outpatient use of people with severe disabilities increased, whereas that of people with mild disabilities who were not eligible for support from the activity assistance policy decreased. This can be interpreted as these disability policies have contributed to improving medical access for people with disabilities.

Considering the fewer programs for people with disabilities and insufficient accessibility, policy support for providing various services is necessary^17^. This study demonstrates that the PAS program has positively impacted medical accessibility for people with disabilities. These findings provide evidence to support the design of customized policies tailored to specific disability types in the future and the expansion of support to include not only individuals with severe disabilities but also those with mild disabilities. This impact is expected to be further validated as the PAS program, since its expansion in 2019, now encompasses individuals with mild disabilities in Korea.

Hypertension is a chronic disease for which lifestyle management, which requires providing various services, is essential^18,19^. Recently, Korea has been running a policy for health doctors for people with disabilities in addition to the Activity Support Program. This project designated a doctor to establish a healthcare plan and provide education and counseling to people with disabilities so that they could continuously and comprehensively manage their health related to chronic diseases and disabilities. Additionally, it provides various medical services, such as managing patients or providing visiting services through non-face-to-face educational counseling if movement is inconvenient. A well-linked activity assistance policy and family doctor system will help improve medical access for people with disabilities and narrow health disparities.

By increasing access to medical care for people with disabilities, these policies can help reduce medical costs. It is difficult for people with disabilities to visit the hospital conveniently owing to various environmental barriers, such as difficulties in moving and the inconvenience of equipment tailored for the non-disabled. This causes a gap in medical expenses between people with and without disabilities. In Korea, the medical expenses for one person with a disability are 3.3 times higher than those for a person without a disability. According to an analysis of U.S. Medical Expenditure Panel Survey data (2004–2008), the annual medical cost for employed people with disabilities was approximately $4,524, approximately $1,325 higher than that of non-people with disabilities^9^. According to Taiwan’s National Health Insurance data analysis, 7.95% of the general public used inpatient treatment, whereas 27.88% of the people with disabilities received inpatient treatment more than three times. Improving chronic disease management by enhancing medical accessibility for people with disabilities can reduce costly inpatient and emergency medical care over time^20^. This, in turn, may lower overall medical expenditures and enhance the efficiency of the healthcare system.

The effects of the PAS program may vary between the short-term and long-term. In the present study, we identified an increase in hypertension-related outpatient visits in the short term, which suggests improved access to healthcare services. This finding aligns with prior research highlighting the importance of inclusive health promotion strategies for people with disabilities, as improved access can help address their heightened risk for secondary conditions^21^. If these increased visits facilitate better management of chronic care needs, it is plausible that the frequency of visits may decrease over time, particularly reducing the need for inpatient care. Should such long-term outcomes be observed, they would offer compelling evidence of the program’s effectiveness. Thus, future research should prioritize investigating the long-term impacts of the PAS program.

In the subgroup analysis, we analyzed whether the policy was effective according to disability type [Supplementary Table 2]. It was found that the type classified as others increased hospital visits after the activity support policy compared to other disabilities. This is because there are no separate categories of disorders other than physical disorders, acquired brain injury, vision impairment, and deafness or hard of hearing (such as electrical disorders, mental disorders, kidney disorders, speech disorders, and automation) in classifying disorders within the NHIS data.

The limitations of this study were as follows. First, it used secondary data from the sample cohort of the Health Insurance Corporation. Therefore, an omitted variable problem may have occurred. Second, the effect of the policy is expected to be more significant because all people with disabilities will be able to use the services by 2019. However, as the data used in the analysis were collected until 2019, this cannot be confirmed. If more data are accumulated, subsequent studies can confirm the effects of expanding targets. Third, Due to limitations in the study data, this research could only examine short-term effects, which is a noted limitation. Finally, this study focused on general medical care for people with disabilities with hypertension. Further studies can narrow it to medical use related to hypertension treatment.

## Conclusion

This study investigated whether the Activity Support Program for people with disabilities was related to hospital visits. The results revealed that expanding enrollment in programs for people with disabilities was linked to an increase in outpatient visits among people with severe disabilities. This study is noteworthy because the expansion of services could potentially help bridge health disparities.

## Electronic supplementary material

Below is the link to the electronic supplementary material.

Supplementary Material 1

## Data Availability

Data can be obtained after application and approval by the National Health Insurance Service.
