# Supplementary material for: Evaluating the impact of disability support services on healthcare utilization in individuals with disabilities and hypertension in Korea
Source: Sci Rep. 2025 Apr 24;15:14388. doi: 10.1038/s41598-025-86915-x (PMC12022007; doi:10.1038/s41598-025-86915-x)
Supplement: Supplementary file 1 — Supplementary Material 1 [file 41598_2025_86915_MOESM1_ESM.docx]

**Supplementary material**

**Table 1. Unadjusted Trend of Hospital Visits Due to Hypertension**

|  | **Total visits** | | | | | **Outpatient visits** | | | | | **Inpatient visits** | | | | |
| --- | --- | --- | --- | --- | --- | --- | --- | --- | --- | --- | --- | --- | --- | --- | --- |
|  | **Severe** | **Annual rate of change** | **Mild** | **Annual rate of change** | **Total** | **Severe** | **Annual rate of change** | **Mild** | **Annual rate of change** | **Total** | **Severe** | **Annual rate of change** | **Mild** | **Annual rate of change** | **Total** |
| **2010** | 15162 |  | 10012 |  | 25174 | 14414 |  | 9779 |  | 24193 | 748 |  | 233 |  | 981 |
| **2011** | 15798 | 4.19 | 11981 | 19.67 | 27783.19 | 14941 | 3.66 | 11590 | 18.52 | 26534.66 | 857 | 14.57219 | 391 | 67.81116 | 1262.572 |
| **2012** | 21799 | 37.99 | 14325 | 19.56 | 36161.99 | 20774 | 39.04 | 13937 | 20.25 | 34750.04 | 1025 | 19.60327 | 388 | -0.76726 | 1432.603 |
| **2013** | 24407 | 11.96 | 16522 | 15.34 | 40940.96 | 23123 | 11.31 | 15936 | 14.34 | 39070.31 | 1284 | 25.26829 | 586 | 51.03093 | 1895.268 |
| **2014** | 27440 | 12.43 | 17674 | 6.97 | 45126.43 | 26028 | 12.56 | 16997 | 6.66 | 43037.56 | 1412 | 9.968847 | 677 | 15.52901 | 2098.969 |
| **2015** | 30802 | 12.25 | 19458 | 10.09 | 50272.25 | 29244 | 12.36 | 18747 | 10.30 | 48003.36 | 1558 | 10.33994 | 711 | 5.022157 | 2279.34 |
| **2016** | 35164 | 14.16 | 22799 | 17.17 | 57977.16 | 32780 | 12.09 | 21701 | 15.76 | 54493.09 | 2384 | 53.01669 | 1098 | 54.43038 | 3535.017 |
| **2017** | 39276 | 11.69 | 25074 | 9.98 | 64361.69 | 36558 | 11.53 | 23994 | 10.57 | 60563.53 | 2718 | 14.01007 | 1080 | -1.63934 | 3812.01 |
| **2018** | 43389 | 10.47 | 29352 | 17.06 | 72751.47 | 40478 | 10.72 | 27956 | 16.51 | 68444.72 | 2911 | 7.100809 | 1396 | 29.25926 | 4314.101 |
| **2019** | 48201 | 11.09 | 31332 | 6.75 | 79544.09 | 44809 | 10.70 | 29814 | 6.65 | 74633.7 | 3392 | 16.52353 | 1518 | 8.739255 | 4926.524 |

**Table 2. Subgroup analysis**

| **Variable** | | | **Average change of total**  **visits** | **Average change of outpatient visits** | **Average change of inpatient visits** |
| --- | --- | --- | --- | --- | --- |
| Intercept | | | 2.083*** (0.102) | 1.970*** (0.109) | 0.146 (0.307) |
| Policy (Ref: Control) | | | 0.092** (0.041) | 0.094** (0.045) | 0.188 (0.119) |
| Type of disability (Ref: Others) | | |  |  |  |
| Physical | | | -0.619*** (0.039) | -0.605*** (0.043) | -0.888*** (0.116) |
| Acquired brain injury | | | -0.509*** (0.062) | -0.628*** (0.067) | -0.843*** (0.171) |
| Vision impairment | | | -0.569*** (0.065) | -0.543*** (0.070) | -1.032*** (0.203) |
| Deaf or difficulties in hearing | | | -0.584*** (0.082) | -0.575*** (0.089) | -0.708** (0.252) |
| Policy*Case (Ref: Control, Others) | | |  |  |  |
| Policy*Physical | | | -0.208*** (0.051) | -0.219*** (0.055) | -0.173 (0.151) |
| Policy*Acquired brain injury | | | -0.185** (0.081) | -0.240** (0.088) | -0.041 (0.226) |
| Policy*Vision impairment | | | -0.147 (0.087) | -0.158 (0.094) | -0.035 (0.269) |
| Policy*Deaf or difficulties in hearing | | | -0.242** (0.112) | -0.236 (0.121) | -0.503 (0.347) |
| Type of healthcare insurance (Ref: Medical Aid) | | |  |  |  |
| Local-subscriber | | | -0.083** (0.030) | -0.041 (0.032) | -0.711*** (0.087) |
| Employer-sponsored | | | -0.081** (0.028) | -0.027 (0.029) | -1.034*** (0.081) |
| Sex (Ref: Male) | | | 0.025 (0.024) | 0.036 (0.026) | -0.107 (0.072) |
| Age | | | 0.010*** (0.002) | 0.011*** (0.002) | 0.004 (0.005) |
| Season (Ref: Winter) | | |  |  |  |
| Spring | | | 1.193*** (0.014) | 1.222*** (0.044) | 0.565*** (0.126) |
| Summer | | | 0.299*** (0.047) | 0.301*** (0.050) | 0.227 (0.143) |
| Autumn | | | 0.198** (0.053) | 0.169** (0.057) | 0.471** (0.160) |
|  |  | * P-values <0.05, <0.01, and <0.001 are denoted using *, **, and ***, respectively. | | | |
